# Fungal siderophore biosynthesis is partially localized in peroxisomes

**DOI:** 10.1111/mmi.12225

**Published:** 2013-04-26

**Authors:** Mario Gründlinger, Sabiha Yasmin, Beatrix Elisabeth Lechner, Stephan Geley, Markus Schrettl, Michael Hynes, Hubertus Haas

**Affiliations:** 1 Division of Molecular Biology/Biocenter, Innsbruck Medical UniversityInnrain 80-82, A-6020, Innsbruck, Austria; 2 Division of Molecular Pathophysiology/Biocenter, Innsbruck Medical UniversityInnrain 80-82, A-6020, Innsbruck, Austria; 3 Department of Genetics, University of MelbourneParkville, 3010, Australia

## Abstract

Siderophores play a central role in iron metabolism and virulence of most fungi. Both *Aspergillus fumigatus* and *Aspergillus nidulans* excrete the siderophore triacetylfusarinine C (TAFC) for iron acquisition. In *A. fumigatus*, green fluorescence protein-tagging revealed peroxisomal localization of the TAFC biosynthetic enzymes SidI (mevalonyl-CoA ligase), SidH (mevalonyl-CoA hydratase) and SidF (anhydromevalonyl-CoA transferase), while elimination of the peroxisomal targeting signal (PTS) impaired both, peroxisomal SidH-targeting and TAFC biosynthesis. The analysis of *A. nidulans* mutants deficient in peroxisomal biogenesis, ATP import or protein import revealed that cytosolic mislocalization of one or two but, interestingly, not all three enzymes impairs TAFC production during iron starvation. The PTS motifs are conserved in fungal orthologues of SidF, SidH and SidI. In agreement with the evolutionary conservation of the partial peroxisomal compartmentalization of fungal siderophore biosynthesis, the SidI orthologue of coprogen-type siderophore-producing *Neurospora crassa* was confirmed to be peroxisomal. Taken together, this study identified and characterized a novel, evolutionary conserved metabolic function of peroxisomes.

## Introduction

Iron is an essential nutrient for all eukaryotes and nearly all prokaryotes (Kaplan and Kaplan, 2009). Moreover, the control over iron access is one of the central battlefields during infection as pathogens have to ‘steal’ the iron from the host – in particular as the mammalian innate immune system restricts iron access to pathogens via a variety of mechanisms (Ganz, 2009; Weinberg, 2009).

*Aspergillus fumigatus* is a ubiquitous saprophytic fungus, which has become the most common air-borne fungal pathogen of humans (Tekaia and Latge, 2005). Clinical manifestations range from allergic reactions to life-threatening invasive disease, termed aspergillosis, particularly in immunocompromised patients. The identification and functional characterization of more than 20 genes that are involved in iron homeostasis maintenance in *A. fumigatus* and/or its less pathogenic relative *Aspergillus nidulans* have made them models for iron metabolism in filamentous fungi (Haas, 2012). Both *Aspergillus* species employ low-affinity ferrous iron acquisition as well as siderophore-assisted iron uptake, a high-affinity ferric iron uptake system (Eisendle *et al*., 2003; Schrettl *et al*., 2004). In contrast to *A. nidulans, A. fumigatus* possesses a second high-affinity iron uptake system, termed reductive iron assimilation. Interestingly, the fungal model system *Saccharomyces cerevisiae* differs from most other fungi due to the lack of siderophore biosynthesis and employment of iron regulators that are not conserved in most other fungal species (Haas *et al*., 2008). Both *Aspergillus* species excrete the fusarinine-type siderophore triacetylfusarinine C (TAFC) to mobilize extracellular iron. Iron-chelated TAFC is taken up by siderophore-iron transporters (Haas *et al*., 2003; 2008; Philpott and Protchenko, 2008). Intracellular release of iron from TAFC involves hydrolysis of the siderophore backbone catalysed in part by the esterase EstB (Kragl *et al*., 2007). Furthermore, both *Aspergillus* species produce the intracellular siderophore ferricrocin (FC) for hyphal iron storage and distribution (Schrettl *et al*., 2007; Wallner *et al*., 2009; Blatzer *et al*., 2011).

**Fig. 1:**
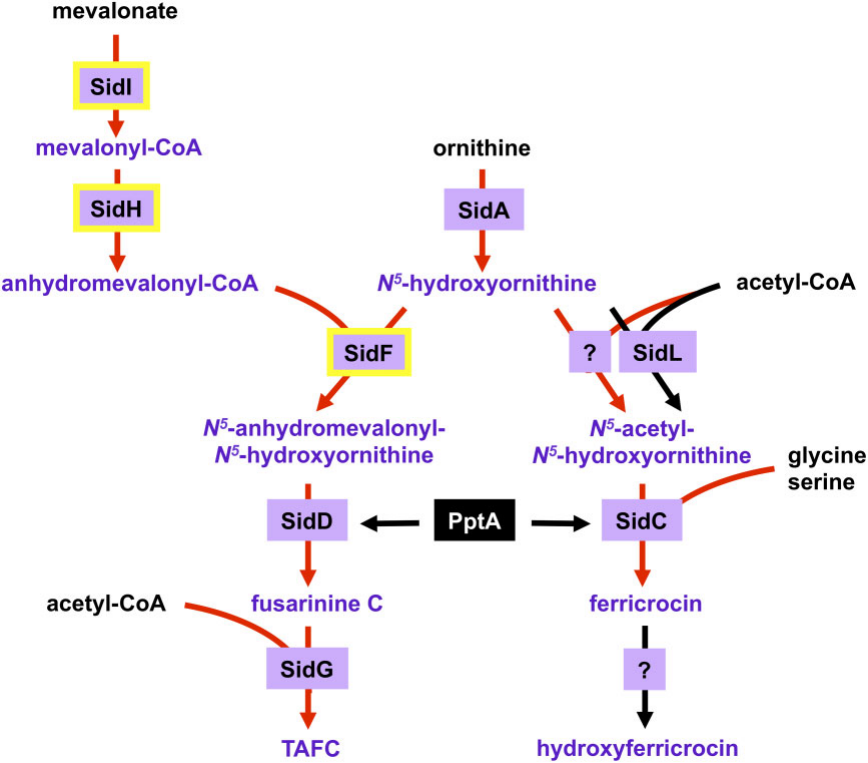
The siderophore biosynthetic pathway of *A. fumigatus* and *A. nidulans*. The enzymes boxed in purple are described in the text. Enzymes transcriptionally upregulated during iron starvation are marked by red arrows. The enzymes identified in this study to localize to peroxisomes are framed in yellow. Adapted from Haas (2012).

TAFC consists of three *N^2^*-acetyl-*N^5^*-anhydromevalonyl-*N^5^*-hydroxyornithine residues cyclically linked by ester bonds; FC is a cyclic hexapeptide with the structure Gly–Ser–Gly–(*N^5^*-acetyl-*N^5^*-hydroxyornithine)_3_ (Haas *et al*., 2008). The siderophore biosynthetic pathway is shown in Fig. 1. The first committed step in the biosynthesis of these siderophores is the hydroxylation of ornithine catalysed by the ornithine monooxygenase SidA (Eisendle *et al*., 2003; Schrettl *et al*., 2004; Olucha *et al*., 2011). Subsequently, the pathways for biosynthesis of extra- and intracellular siderophores split. For biosynthesis of extracellular siderophores, the transacylase SidF transfers anhydromevalonyl to *N^5^*-hydroxyornithine (Schrettl *et al*., 2007). The required anhydromevalonyl-CoA moiety is derived from mevalonate by CoA ligation and dehydration catalysed by SidI and SidH respectively (Yasmin *et al*., 2011). The acetylation of *N^5^*-hydroxyornithine for FC biosynthesis involves two transacetylases, the constitutively expressed SidL (Blatzer *et al*., 2011) and an unidentified enzyme, the activity of which is upregulated by iron starvation. Assembly of Fusarinine C (FsC) and FC is catalysed by two different non-ribosomal peptide synthetases (NRPS), SidD and SidC respectively. Subsequently, SidG catalyses *N^2^*-acetylation of FsC for forming TAFC. As typical for NRPS, SidD and SidC depend on activation by 4′-phosphopantetheinyl transferase (Oberegger *et al*., 2003). Both extra- and intracellular siderophores are crucial for growth during iron limitation in *A. fumigatus* and *A. nidulans* (Eisendle *et al*., 2003; Schrettl *et al*., 2004). Elimination of the entire siderophore biosynthesis (Δ*sidA* mutant) results in absolute avirulence of *A. fumigatus* in a murine model of invasive pulmonary aspergillosis (Schrettl *et al*., 2004; Hissen *et al*., 2005), while deficiency in either extracellular (*ΔsidI*, *ΔsidH*, *ΔsidF* or *ΔsidD* mutants) or intracellular siderophores (*ΔsidC* mutants) causes partial attenuation of virulence (Schrettl *et al*., 2007; Yasmin *et al*., 2011).

Peroxisomes are single membrane organelles, which compartmentalize a wide range of metabolic functions in eukaryotic cells. Important functions of peroxisomes include fatty acid β-oxidation, the glyoxylate cycle, metabolisms of cholesterol and reactive oxygen species, as well as methanol oxidation (Schrader and Fahimi, 2008). Due to the diversity of metabolic pathways in peroxisomes, their content varies depending on the species, cell or tissue type, as well as on environmental conditions (Brown and Baker, 2008). In contrast to mitochondria, peroxisomes are devoid of DNA and protein synthesis machinery. Therefore, all peroxisomal proteins are encoded by nuclear genes and are post-translationally imported into peroxisomes. In *S. cerevisiae*, more than 60 peroxisomal proteins have been identified including the peroxins (Pex), which are essential for peroxisomal biogenesis and maintenance as well as matrix protein import (Schrader and Fahimi, 2008).

This study revealed peroxisomal targeting signal of type one (PTS1) or type two (PTS2) in SidI, SidH and SidF of *Aspergillus* spp. and their orthologues in other *Ascomycetes*. Peroxisomal localization of all three enzymes was confirmed by green fluorescence protein (GFP) tagging and by PTS1 mutation of SidH in *A. fumigatus*. The analysis of *A. nidulans* mutants deficient in peroxisomal biogenesis, ATP import and protein import depending on either PTS1, PTS2, or both, indicated that cytosolic mislocalization of individual enzymes but not of the entire TAFC biosynthetic machinery impairs TAFC production during iron starvation.

## Results

### *A. fumigatus* SidI, SidH and SidF carry PTS and localize to peroxisomes

The majority of peroxisomal matrix proteins are post-translationally directed to the lumen of the organelle by peroxisomal targeting signals (PTS) and two different motifs have been characterized (Olivier and Krisans, 2000). PTS1 is a tri-peptide with the consensus sequence (S/A/C)(K/H/R)(L/M) located at the extreme C-terminus, whereas PTS2 is a nona-peptide of the consensus sequence (R/K)(L/V/I)X_5_(H/Q)(L/A) located near the N-terminus of a matrix protein. Sequence analysis of the *A. fumigatus* TAFC biosynthetic enzymes revealed the presence of the putative PTS1 motifs SKL and AKL in SidH and SidF, respectively, and a putative PTS2, RLQTLSQHL, localized at amino acid 6–14 in SidI.

**Fig. 2:**
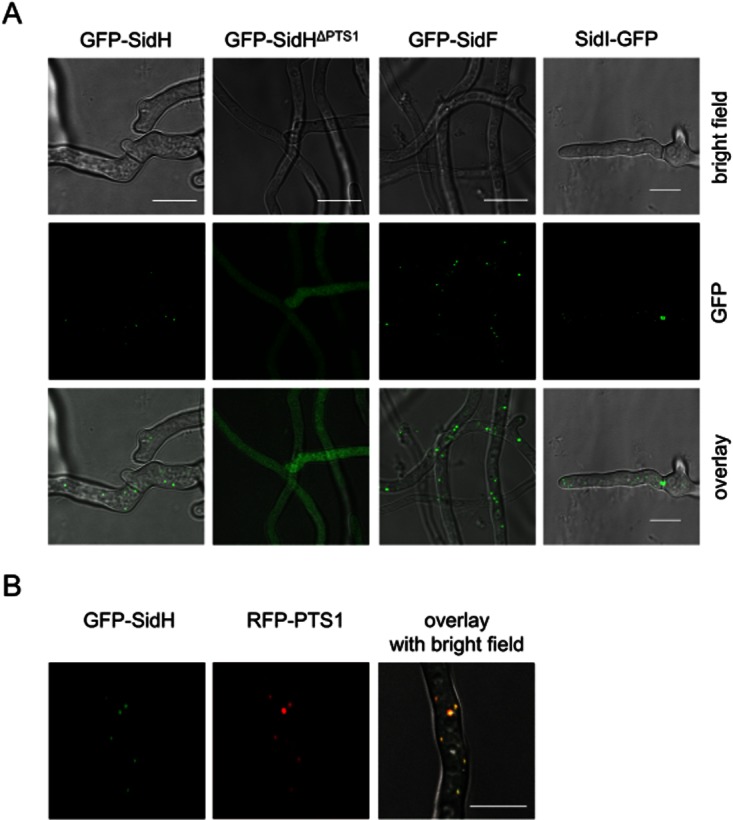
A. In *A. fumigatus,* GFP-tagged versions of SidH (GFP–SidH), SidF (GFP–SidF) and SidI (SidI–GFP) localize to peroxisomes while PTS1 truncation (GFP–SidH^ΔPTS1^) blocks peroxisomal localization of SidH. B. GFP-tagged SidH (GFP–SidH) colocalizes with peroxisomal RFP (RFP–PTS1). Fungal strains were grown in iron-depleted minimal medium for 18 h at 37°C. Scale bar, 10 μm.

To confirm the peroxisomal localization of SidH and SidF, *ΔsidH* and *ΔsidF* mutant strains were complemented with respective N-terminally GFP-tagged versions as described in *Experimental procedures*. SidI was C-terminally GFP-tagged in a wild-type (*wt*) strain via integration of the GFP-encoding gene at the *sidI* locus. Consequently, the respective mutant produces only GFP-tagged SidI. Consistent with the predicted peroxisomal localization, the GFP-tagged versions of all three enzymes localized to punctate dots in the cytoplasm (Fig. 2A). *ΔsidH* and *ΔsidF* mutant strains lack TAFC production (Schrettl *et al*., 2007), while the expression of the respective GFP-fusion proteins increased TAFC production to 86 ± 11% and 91 ± 8%, respectively, of the wild type (*wt*). Similarly, the SidI–GFP carrying strain displayed 95 ± 19% of the *wt* TAFC production. These data indicate correct enzymatic activity and subcellular localization of the GFP-tagged protein versions. Truncation of the putative PTS1 motif of GFP–SidH (GFP–SidH^ΔPTS1^) caused cytosolic localization, which strongly suggests that the C-terminus of SidH is indeed a targeting sequence required for peroxisomal localization (Fig. 2A). In contrast to GFP–SidH, GFP–SidH^ΔPTS1^ did not support TAFC synthesis, i.e. TAFC biosynthesis was not detectable in the respective mutant strain (data not show), indicating that peroxisomal localization of SidH is essential for TAFC biosynthesis.

Recently, red fluorescence protein (RFP) C-terminally tagged with a PTS1 motif (RFP–PTS1) has been shown to localize to peroxisomes in *A. fumigatus* (Beck and Ebel, 2013). In a strain producing both GFP–SidH and RFP–PTS1, we found virtually perfect colocalization (Fig. 2B). These data underline the peroxisomal localization of SidH.

### Peroxisomal targeting signals are conserved in fungal SidI, SidH and SidF orthologues

With few exceptions such as *S. cerevisiae*, *Candida albicans* or *Cryptoccoccus neoformans,* most fungi produce siderophores and encode respective genes (Yasmin *et al*., 2011). Genome mining by blast searches and sequence analysis revealed that putative SidI, SidH and SidF orthologues from various *Ascomycetes* carry putative PTS motifs. Table 1 lists the closest homologues to SidI, SidH and SidF, which were identified by blast searches. Genes involved in siderophore biosynthesis (including *sidI*, *sidH* and *sidF*) tend to be organized in gene clusters (Schrettl *et al*., 2008). The orthologues of *sidI, sidH* and *sidF* listed in Table 1 are colocalized in the genome with other putative siderophore biosynthetic gene (Table S1), which emphasizes that these genes are indeed the orthologues. Besides these orthologues, all analysed species possess additional homologues (see below). All identified SidF and SidH orthologues possess PTS1 motifs. Importantly, SidI orthologues from *Sordariomycetes* such as *Neurospora crassa* possess a PTS1 in contrast to the PTS2 found in other *Ascomycetes*.

**Table 1:** Peroxisomal targeting signals in orthologues of SidI, SidH and SidF

		SidI			SidH			SidF		
				
Organism		Accession	PTS	Score (PTS1)	Accession	PTS	Score (PTS1)	Accession	PTS	Score (PTS1)
*Eurotiomycetes*	*Aspergillus fumigatus*	XP_753087.1	RLQQTLSHL	–	XP_748661.1	SKL	10.8	XP_748660.1	AKL	7.5
	*Neosartorya fischeri*	XP_001264038.1	RLQQTLSHL	–	XP_001259092.1	SKL	10.5	XP_001259091.1	AKL	7.5
	*Aspergillus niger*	XP_001390954.1	RLQQTLSHF	–	XP_001390237.1	SKL	9.6	XP_001390236.1	AKL	6.9
	*Aspergillus clavatus*	XP_001268556.1	RLQQTLSHL	–	XP_001273567.1	SNL	9.3	XP_001273568.1	AKL	7.3
	*Aspergillus oryzae*	XP_001821069.1	RLQQTLSHI	–	XP_001826764.1	SNL	5.4	XP_001826765.1	AKL	5.2
	*Penicillium chrysogenum*	XP_002569340.1	RLQQTLSHV	–	XP_002565937.1	SKL	8.1	XP_002565938.1	AKL	6.9
	*Aspergillus nidulans*	XP_658213.1	RLQQTLSHL	–	XP_663839.1	SNL^d^	5.9	XP_663838.1	AKL	7.7
	*Ajellomyces capsulatus*	EEH02760.1	RLQQTLNHI	–	EEH07298.1	SKL	12.4	XP_001536540.1	SKL	14.0
	*Talaromyces stipitatus*	XP_002484634	RLQQTQRHI	–	XP_002486020.1	SKL	8.4	XP_002486021.1	AKL	6.9
*Leotiomycetes*	*Botryotinia fuckeliana*	XP_001546797.1	PKL^a^	13.5	XP_001551006	SKL	9.1	XP_001551005.1	PKL^a^	6.5
	*Sclerotinia sclerotiorum*	XP_001585101.1	PKL	12.7	XP_001594442.1	SKL^b^	9.1	XP_001594441.1	AKL	6.9
*Sordariomycetes*	*Neurospora crassa*	XP_959826.1	SKL	6.2	XP_962600.1	SKL	6.3	XP_959825.1	PKL	6.5
	*Gibberella zeae*	XP_384509.1	SKL	3.7	XP_383922.1	SKL	10.4	XP_383921.1	PKL	2.2
	*Podospora anserina*	XP_001905332.1	AKL	12.6	XP_003437360.1	SKL	8.6	XP_001905331.1	PKL	8.0
	*Chaetomium globosum*	XP_001226170.1	AKL	0.0	XP_001227399.1	SKL	8.6	XP_001227400.1	AKL	6.9
	*Magnaporthe oryzae*	EHA55987	AKL	10.1	EHA47011.1	SKL	9.3	XP_362759.1	PKL	8.2
*Dothideomycetes*	*Phaeosphaeria nodorum*	XP_001804551.1	RLNQTLLQI^c^	–	XP_001790987.1	SKL	11.7	XP_001804552.1	ARL	8.5
*Basidiomycota*	*Ustilago maydis*	XP_760950.1	–	–	XP_757580.1 *F*er4	–	–	XP_757579.1 Fer5	–	–

a Updated genome data from *Botrytis cinerea* strain B05.10 with the locus tag B0510_2726 (SidI) and B0510_7543 (SidF) reveal gene products containing a PTS1 (PKL). http://www.broadinstitute.org/annotation/genome/botrytis_cinerea/FeatureSearch

b The annotated gene is organized in four exons, whereas all other orthologues have two. Manual reannotating of the intron/exon structure increased the sequence similarity to its orthologues and led to a PTS1-containing C-terminus (EWYPRLVKSPNFAEGIQAYVDKRPPKWVNSKL).

c The correct start codon is most likely 414 bp upstream and in frame of the annotated start, which leads to a gene product with higher similarity to its orthologues and contains the quoted PTS2.

d The deposited sequence most likely contains a sequencing error leading to a false C-terminus. Contig 1.106, which was used for gene assembly, misses in contrast to contig 1.107 a cytosine after nt 745 of the cds. Correction of the sequence generated a C-terminus showing 80% identity with *A. fumigatus* SidH (EEASSALVDEWYPKLIAGENFHEGVKAFVEKRQPRWRASNL).

Variants of the classical PTS1 SKL sequence such as –ARL, –AKL or –PKL were shown to be functional peroxisomal targeting signals in human, yeast and *Penicillium chrysogenum* (Amery *et al*., 1998; Kiel *et al*., 2009). The PTS1 scores of proteins were obtained using the PTS1-predictor program http://mendel.imp.ac.at/mendeljsp/sat/pts1/PTS1predictor.jsp (Neuberger *et al*., 2003). Positive scores indicate high probability of peroxisomal targeting, sequences with scores < −10 are unlikely to function as PTS1, and motifs with scores in between have unclear function. PTS2 motifs were identified using the PTS2 finder http://www.peroxisomedb.org/diy_PTS2.html, which does not provide reliability scores.

### *A. fumigatus* SidH and SidI are targeted to peroxisomes via their corresponding PTS1 and PTS2 receptors PexE and PexG, respectively, in *A. nidulans*

*Aspergillus fumigatus* and *A. nidulans* produce the same siderophores and the proteins involved in their biosynthesis are highly conserved (Haas *et al*., 2008; Schrettl *et al*., 2008). The amino acid sequence identities of the corresponding TAFC biosynthetic enzymes of *A. fumigatus* and *A. nidulans* are given in Table S2. Moreover, the PTS are perfectly conserved (Table 1). To investigate the peroxisomal localization of TAFC biosynthesis in more detail, we switched from *A. fumigatus* to *A. nidulans,* because the peroxisomal system of the latter has been characterized in great detail and a number of respective peroxin mutants, described in Table S3, is available (Hynes *et al*., 2008). Peroxins are proteins required for the assembly and function of peroxisomes. These are highly conserved among fungal species (Kiel *et al*., 2006); a comparison of relevant peroxins of *A. fumigatus* and *A. nidulans* is given in Table S3 (Hynes *et al*., 2008).

**Fig. 3:**
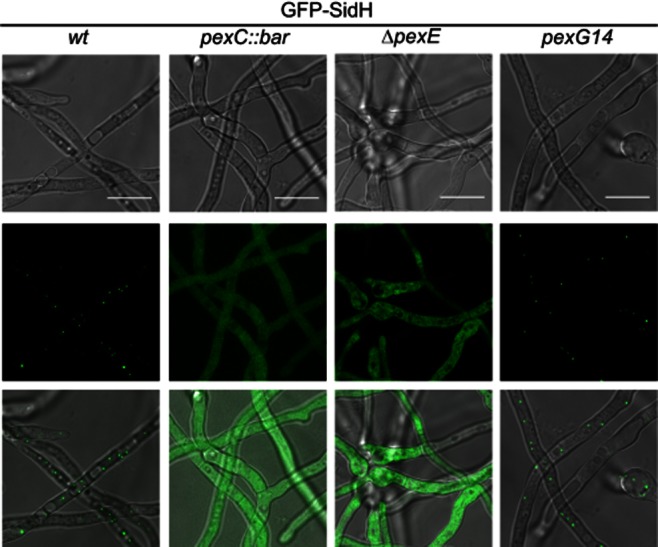
In *A. nidulans*, peroxisomal localization of *A. fumigatus* GFP–SidH is blocked by inactivation of PexC (*pexC::bar*) or PexE (*ΔpexE*) but not PexG (*pexG14*). Fungal strains were grown in iron-depleted minimal medium for 18 h at 37°C. Upper panel, bright-field image; mid-panel, confocal fluorescence microscopy; lower panel, overlay. Scale bar, 10 μm.

In a first step, localization of *A. fumigatus* GFP–SidH was heterologously studied in *A. nidulans wt* and mutants lacking peroxisomes (*pexC::bar*), PTS1-dependent peroxisomal import (*ΔpexE,* missing the PTS1 receptor PexE) or PTS2-dependent peroxisomal targeting (*pexG14,* lacking a functional PTS2 receptor PexG). In perfect agreement with PTS1 receptor-mediated peroxisomal localization, GFP–SidH localized in cytosolic spots in *A. nidulans wt* and *pexG14* strains but mislocalized to the cytosol in *ΔpexE* and *pexC::bar* mutants (Fig. 3). The peroxisomal localization of GFP–SidF in *A. fumigatus* together with its evolutionary conserved PTS1 (Fig. 2, Table 1) strongly suggests that SidF is directed to peroxisomes in the same manner.

**Fig. 4:**
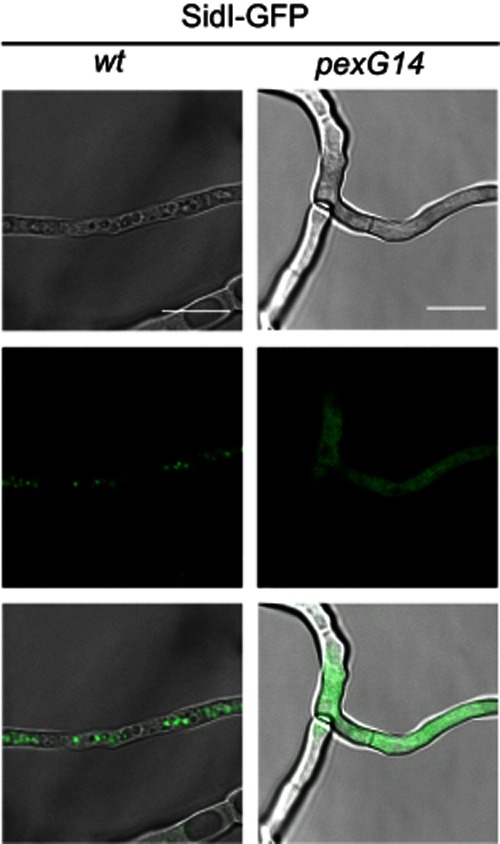
In *A. nidulans*, peroxisomal localization of *A. fumigatus* SidI–GFP is blocked by inactivation of PexG (*pexG14*). Fungal strains were grown in iron-depleted minimal medium containing 200 μM BPS to generate harsh iron starvation for 24 h at 37°C. Upper panel, bright-field image; mid-panel, confocal fluorescence microscopy; lower panel, overlay. Scale bar, 10 μm.

In contrast to GFP–SidH, SidI–GFP was mislocalized to the cytosol in a *pexG14* strain missing the PTS2 receptor, which demonstrates a PTS2-dependent peroxisomal targeting of SidI (Fig. 4).

These data confirm that PTS1 and PTS2 receptors function independently in filamentous fungi, which contrasts with the situation in mammals and plants (Kiel *et al*., 2006; Hynes *et al*., 2008). Moreover, the data suggest that inactivation of the PTS1 receptor mislocalizes SidH and SidF, while inactivation of the PTS2 receptor mislocalizes SidI to the cytosol.

### Deficiency in either PTS1- or PTS2-dependent peroxisomal protein import or AntA-mediated peroxisomal ATP import decreases TAFC production in *A. nidulans*

**Fig. 5:**
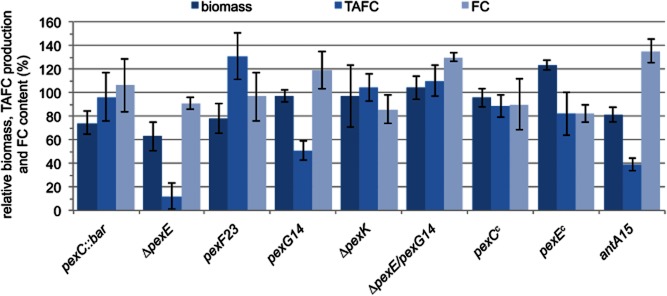
Inactivation of PexE, PexG or AntA but not of PexC, PexK or both PexE and PexG impairs TAFC biosynthesis. Production of TAFC and FC was measured after growth for 24 h in iron-depleted minimal medium. The values represent the means ± STD of three experiments normalized to biomass and *wt* (100%).

To characterize the role of peroxisomes in siderophore biosynthesis at the metabolite level, production of TAFC and FC was analysed in the *A. nidulans wt* and mutants affected in peroxisomal import (*ΔpexE, pexG14, pexF23, ΔpexE/pexG14*), peroxisomal proliferation (*ΔpexK*), peroxisomal biogenesis (*pexC::bar*) or peroxisomal ATP import (*antA14*) respectively (Fig. 5).

In accordance with the importance of PexE-mediated peroxisomal transport of SidH and SidF, the TAFC production of the *pexE* deletion mutant (*ΔpexE*) was decreased to 12% of the *wt* during iron starvation (Fig. 5). The decreased TAFC production of *ΔpexE* is most likely the reason for the 37% reduced biomass production compared with *wt* (Fig. 5) because the lack of siderophore production has been shown to decrease growth during iron limitation (Schrettl *et al*., 2004; 2007). The TAFC and biomass production defects during iron starvation are cured by *pexE* gene complementation in strain *pexE^c^* (Fig. 5). Inactivation of PexG-mediated peroxisomal import (strain *pexG14*) reduced TAFC production to 51% of the *wt*, which suggests a crucial role for PTS2-dependent peroxisomal import of SidI in TAFC biosynthesis. In agreement with the higher TAFC production compared with *ΔpexE*, pexG14 biomass production was also higher. Taken together, these data indicate that cytosolic mislocalization of SidF and SidH or of SidI via inactivation of PTS1 or PTS2 receptors, respectively, impairs TAFC biosynthesis in *A. nidulans*.

The peroxisomal membrane is impermeable to ATP (Palmieri *et al*., 2001; van Roermund *et al*., 2001; Hynes *et al*., 2008). In *A. nidulans,* inactivation of the peroxisomal ATP-importer AntA in the *antA15* strain reduces growth on fatty acids (Hynes *et al*., 2008) because ATP is required to fuel peroxisomal β-oxidation by activation of fatty acyl-CoA synthetases, which belong to the acyl-CoA-ligase family. SidI belongs also to the acyl-CoA ligase protein family and converts mevalonate to mevalonyl-CoA in an ATP-dependent manner (Yasmin *et al*., 2011). Consistent with the requirement for ATP within peroxisomes for SidI activity, TAFC production was reduced to 39% of *wt* in *antA15*, a similar reduction to that observed for *pexG14*. Both β-oxidation and TAFC biosynthesis were not blocked completely in *antA15* suggesting an AntA-independent peroxisomal ATP delivery system. In this respect it can be noted that glycolytic/gluconeogenic enzymes have recently been shown to have dual localization in fungi, i.e. in the cytoplasm and peroxisomes (Freitag *et al*., 2012). This metabolic network may function in redox/ATP shuttling or as a buffer system to cope with perturbations of redox/ATP equivalents (Freitag *et al*., 2012).

Remarkably, neither blocking of peroxisome biogenesis (*pexC::bar*) nor inactivation of entire peroxisomal protein import by simultaneous inactivation of both PexE and PexG (Δ*pexE/*pexG14), or inactivation of pexF (*pexF26*) impaired TAFC production (Fig. 5). Remarkably, the TAFC production of *pexF26,* which lacks PexF and consequently both PTS1- and PTS2-dependent peroxisomal protein targeting (Erdmann and Schliebs, 2005; Koek *et al*., 2007; Hynes *et al*., 2008), was even 31% increased compared with *wt* (Fig. 5). Peroxisomes are generated either *de novo* from the endoplasmatic reticulum, which requires PexC, or by PexK-dependent peroxisomal division of the novo-generated peroxisomes (Hoepfner *et al*., 2005; Hettema and Motley, 2009). PexK deficiency (*ΔpexK*) also did not affect TAFC production (Fig. 5).

The FC content of *ΔpexE, pexF23, pexC::bar* and *ΔpexK* was between 85% and 105% of the *wt* indicating that FC biosynthesis does not rely on peroxisomes (Fig. 5). In agreement, SidI, SidH and SidF are not involved in FC biosynthesis (Fig. 1). Interestingly, FC production was increased between 19% and 35% compared with *wt* in *pexG14*, *ΔpexE/*pexG14 and *antA15*.

### Inactivation of PexE, PexG or AntA impairs growth under iron-depleted conditions

**Fig. 6:**
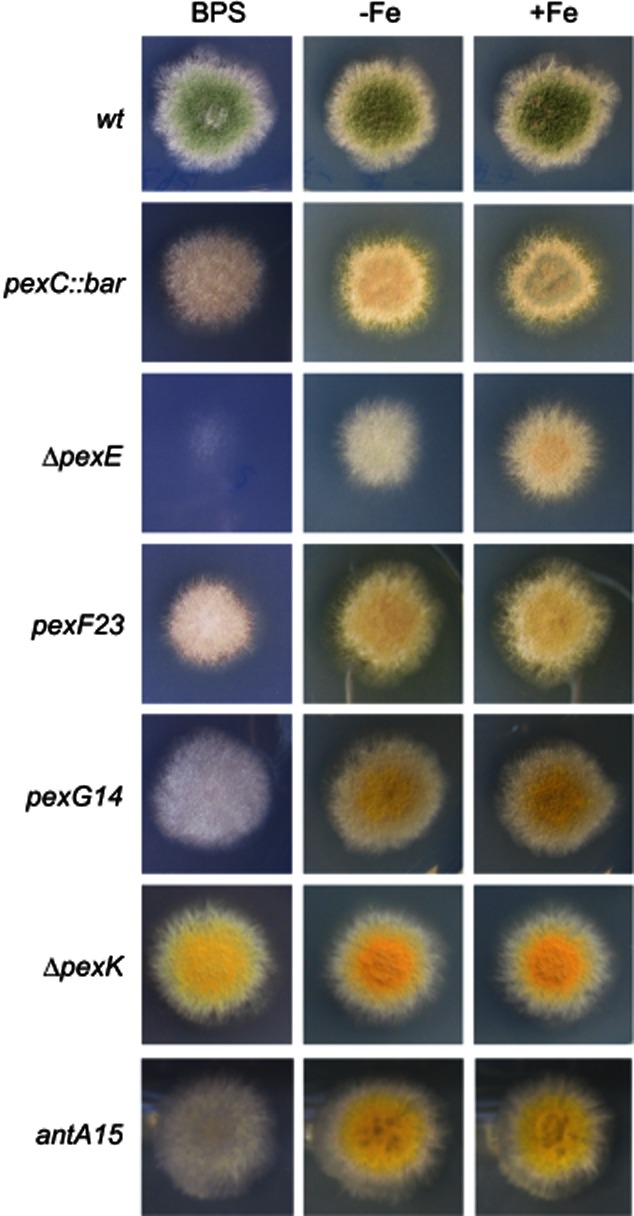
Growth phenotypes of *A. nidulans wt* and peroxisomal mutants. Fifty conidia of the fungal strains were point-inoculated onto minimal medium plates with different iron supply (BPS, 200 μM BPS; −Fe, without addition of iron; +Fe, sufficient iron supply with 30 μM FeSO_4_) and incubated for 48 h. BPS is a chelator generating harsh iron starvation. The *wt* produces green conidia, while the mutant strains produce yellow conidia, due to the genetic marker yA1.

**Fig. 7:**
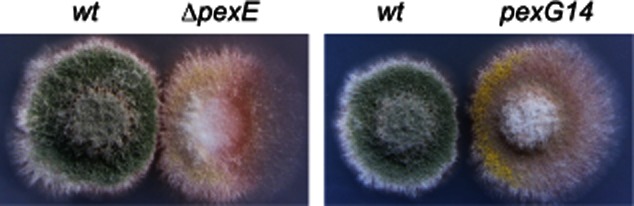
In *A. nidulans*, cross-feeding from *wt* cures the growth and sporulation defects of *ΔpexE* and *pexG14* respectively. Fifty conidia of the *wt* and respective mutant strain were spotted in near distance onto minimal medium agar containing 200 μM BPS to generate harsh iron starvation and incubated for 48 h.

Consistent with the extremely reduced TAFC production and biomass production in liquid culture (Fig. 5), PexE deficiency dramatically decreased growth on plates under iron starvation but not iron sufficiency (Fig. 6). All mutants lacking peroxisomes or PTS1-dependent peroxisomal protein import display reduced conidiation (Hynes *et al*., 2008). However, deficiency in PexG or AntA completely blocked sporulation during iron starvation, most likely due to the decreased TAFC production because siderophore-mediated iron supply has previously been shown to be crucial for sporulation (Schrettl *et al*., 2007; Wallner *et al*., 2009). Consistently, siderophore cross-feeding from *wt* increased growth and conidiation of *ΔpexE* and *pexG14* (Fig. 7).

### The *N. crassa* SidI orthologue localizes to peroxisomes in *Aspergillus* spp. and assists TAFC biosynthesis

**Fig. 8:**
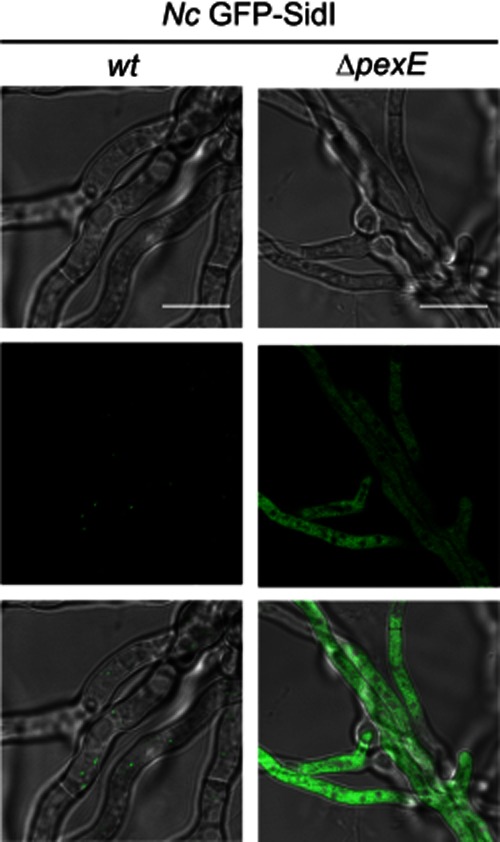
Peroxisomal localization of *N. crassa* GFP–SidI in *A. nidulans* is PexE-dependent. Fungal strains were grown in iron-depleted minimal medium for 18 h at 37°C. Upper panel, bright-field image; mid-panel, confocal fluorescence microscopy; lower panel, overlay. Scale bar, 10 μm.

In contrast to *A. fumigatus* and *A. nidulans*, *N. crassa* secretes the siderophore coprogen instead of TAFC, but shares the same hyphal siderophore FC (Matzanke *et al*., 1987). Similar to TAFC, coprogen contains anhydromevalonyl residues (Haas *et al*., 2008). Therefore, it is not surprising that *N. crassa* possesses orthologues to SidH, SidF and SidI. The SidI orthologues from *A. fumigatus* and *N. crassa* share 60% identity at the amino acid sequence level, but in contrast to the PTS2-carrying *A. fumigatus* SidI, *N. crassa* SidI harbours a PTS1 motif (Table 1). To confirm its role in siderophore biosynthesis and its peroxisomal localization, the *N. crassa sidI* was expressed as an N-terminal GFP-tagged protein in *A. nidulans wt* and the *A. nidulans ΔpexE* strain (Fig. 8). The *N. crassa* GFP–SidI localized to peroxisomes in *A. nidulans wt,* which emphasizes conservation of peroxisomal localization. In accordance with the PTS1 motif, it was mislocalized to the cytoplasm in *A. nidulans ΔpexE*. Remarkably, the expression of *N. crassa* GFP–SidI increased TAFC production from 11% of the parental *ΔpexE* strain to 86% of the *wt*. In this strain, GFP–SidI, SidH and SidF are now all cytosolic, which clearly shows that colocalization of all three enzymes, either in peroxisomes or in the cytosol, is essential for efficient TAFC production.

## Discussion

This study has revealed peroxisomal localization of the three TAFC biosynthetic enzymes from *A. fumigatus*: PTS1-containing SidH and SidF as well as PTS2-containing SidI. All other identified components of the siderophore biosynthetic machinery lack PTS motifs indicating cytosolic localization. However, peroxisomal localization of other siderophore biosynthetic enzymes via unidentified targeting sequences, unidentified import systems or cryptic PTS motifs that are unmasked after post-transcriptional processes cannot be completely excluded (Freitag *et al*., 2012). Nevertheless, the acetyl transferase SidL, required for FC biosynthesis, as well as the esterase EstB, which hydrolyses TAFC after uptake, have been shown to be cytosolic (Kragl *et al*., 2007; Blatzer *et al*., 2011). Cytosolic mislocalization of one to two of the three peroxisomal TAFC biosynthetic enzymes due to PTS1 truncation of SidH in *A. fumigatus* or blocking either PTS1- (strain *ΔpexE*) or PTS2-dependent (strain *pexG14*) peroxisomal protein import in *A. nidulans*, which employs the same siderophore biosynthetic machinery, blocked or at least decreased TAFC biosynthesis. Moreover, blocking peroxisomal ATP import (strain *antA14*), which is required for SidI function, decreased TAFC production in *A. nidulans*. These data suggest that the siderophore pathway intermediates synthesized by SidI and SidH, mevalonyl-CoA and anhydromevalonyl-CoA, cannot efficiently pass the peroxisomal membrane (Fig. 1). Furthermore, the peroxisomal localization of SidI, SidH and SidF indicates that TAFC biosynthesis requires peroxisomal import of *N^5^*-hydroxyornithine and peroxisomal export of *N^5^*-anhydromevalonyl-*N^5^*-hydroxyornithine. Peroxisomal membranes are likely to be permeable to molecules with a molecular mass (*M_r_*) < 400 via channels/pores (Antonenkov *et al*., 2009). Consequently, it is likely that the siderophore precursors mevalonate (*M_r_* = 148) and *N^5^*-hydroxyornithine (*M_r_* = 148) can enter and the SidF product *N^5^*-anhydromevalonyl-*N^5^*-hydroxyornithine (*M_r_* = 260) can freely pass the peroxisomal membrane. In contrast, the CoA-intermediates mevalonyl-CoA (*M_r_* = 916) and anhydromevalonyl-CoA (*M_r_* = 898) are unlikely to be able to freely exit peroxisomes. This feature of the peroxisomal membrane is expected to increase the local concentration of the CoA-intermediates of TAFC biosynthesis, which might enhance the efficiency of the involved enzymatic reactions and consequently provides a rational for the localization in peroxisomes. The decrease in TAFC biosynthesis varied between the different ways of cytosolic mislocalization and can be explained by the different, in part pleiotropic, effects: SidH PTS1 truncation mislocalizes only SidH, PTS1 inactivation mislocalizes SidH and SidF together with all other PTS1-dependent peroxisomal proteins, and PTS2 inactivation mislocalizes SidI together with all PTS2-dependent peroxisomal proteins. Moreover, differences in peroxisomal import and export efficiency of *N^5^*-hydroxyornithine and *N^5^*-anhydromevalonyl-*N^5^*-hydroxyornithine might play a role. In this respect it is interesting to note that the vast majority of peroxisomal matrix proteins are PTS1 receptor-dependent and SidI is one of the few exceptions (Kiel *et al*., 2006). Despite the clear peroxisomal localization of SidI, SidH and SidF, it cannot be excluded that low levels of these enzymes are present and operate in the cytosol.

In agreement with the dramatically reduced TAFC production, the PexE-deficient mutant displayed reduced growth during iron starvation on solid and in liquid growth media (Figs 5 and 6). However, growth of mutants lacking PexC or PexF was also somehow reduced despite their TAFC production not being reduced. These data indicate that siderophore-independent peroxisomal functions are additionally important for adaptation to iron starvation.

Orthologues of SidI, SidH and SidF are found in numerous *Ascomycetes* as these enzymes are not only required for fusarinine-type but also coprogen-type siderophores, which also contain anhydromevalonyl moieties (Haas *et al*., 2008). Genome mining indicated PTS motif conservation in SidI, SidH and SidF orthologues of numerous *Ascomycetes* (Table 1). In agreement, the SidF orthologue of the coprogen producer *Penicillium chrysogenum* was recently identified as a peroxisomal matrix protein in a proteomic approach (Kiel *et al*., 2009). Interestingly, SidI orthologues of *Sordariomycetes* possess a PTS1 in contrast to the PTS2 motif found in other *Ascomycetes* and the PTS1 motif of the SidI orthologue from coprogen-producing *N. crassa* was confirmed to be functional here. Therefore, all three siderophore enzymes in *Sordariomycetes* rely on PexE-dependent peroxisomal import only.

The enoyl-CoA hydratase Fer4 (UMO1433) and the SidF orthologue Fer5 (UM01432.1), which are essential for ferrichrome A biosynthesis in the *Basidiomycete Ustilago maydis* (Winterberg *et al*., 2010)*,* lack PTS indicating non-peroxisomal localization (Table 1). Due to the differences in siderophore structure and biosynthesis, *U. maydis* lacks a SidI orthologue. A BlastP search (http://www.ncbi.nlm.nih.gov/sutils/genom_table.cgi?organism=fungi) identified SidF homologues in various *Basidiomycetes* besides *U. maydis* (Table S7; *Malassezia globosa*, *Serpula lacrymans, Schizophyllum commune, Puccinia graminis, Melampsora larici-populina*) but none of these proteins contained PTS1 or PTS2 motifs, which suggests non-peroxisomal localization of siderophore biosynthesis in *Basidiomycetes*. Moreover, BlastP searches (http://fungidb.org/fungidb/) failed to identify homologues to SidF or SidA in *Chytridiomycetes* (*Batrachochytrium dendrobatidis*)*, Oomycetes* (*Hyaloperonospora arabidopsis, Phytophthora infestans, Pythium ultimum*) or *Zygomyctes* (*Rhizopus oryzae*)*,* which indicates the inability of these species to produce hydroxamate-type siderophores.

Nevertheless, the evolutionary conservation of peroxisomal localization of siderophore biosynthetic enzymes in *Ascomycetes* indicates its importance. Therefore, it is remarkable that cytosolic mislocalization in *A. nidulans* of all three TAFC biosynthetic enzymes by (i) inactivation of peroxisomal biogenesis (strain *pexC::bar*), (ii) simultaneous inactivation of both PTS1- and PTS2- dependent transport (strains *pexG14/ΔpexE and ΔpexF*), or (iii) expression of PTS1-containing SidI from *N. crassa* in a mutant lacking PTS1-dependent peroxisomal protein import (strain GFP–SidI*ΔpexE*) did not decrease TAFC biosynthesis.

Taken together these data suggest that siderophore biosynthesis can efficiently work in both peroxisomes and the cytosol as long as SidI, SidH and SidF share the same compartment. In contrast, peroxisomes are essential for biotin biosynthesis in *A. nidulans*, because it has originally been shown that *pex* mutants defective in PTS1 protein import were found to be auxotrophic for biotin due to an inability to synthesize the intermediate pimelic acid (Hynes *et al*., 2008). Further studies confirmed peroxisomal localization of BioF, a biotin synthetic enzyme in *A. nidulans* and *A. oryzae* (Magliano *et al*., 2011; Tanabe *et al*., 2011). Peroxisomes are indispensible for Ak toxin biosynthesis in the fungal plant pathogen *Alternaria alternate* (Imazaki *et al*., 2010) and β-oxidation in *A. nidulans* (Hynes *et al*., 2008). Similarly, cytosolic mislocalization of the single peroxisomal penicillin biosynthetic enzyme completely blocks penicillin biosynthesis in *P. chrysogenum* (Muller *et al*., 1992). Nevertheless, there are known pathways that are localized in peroxisomes but also work outside. For example, the acyl-CoA transferase IAT (containing a PTS1 motif), which is involved in penicillin biosynthesis, works better in peroxisome but is still functional in the cytosol in *A. nidulans* (Sprote *et al*., 2009). Moreover, in the glyoxylate cycle both malate synthase (using acetyl-coA and glyoxylate) and isocitrate lyase (producing glyoxylate and succinate) normally operate in peroxisomes, but cytoplasmic localization of both allows growth on acetate (Hynes *et al*., 2008). These data raise the general question for the rationale of peroxisomal localization of pathways that are also functional outside. A possible explanation is that all the mentioned pathways include CoA-ligands and peroxisomes appear to provide the optimal conditions for this, which might be favoured by the distinct permeability feature of the peroxisomal membrane mentioned above and the high local CoA concentration. Another possible explanation for the siderophore biosynthetic enzymes could be their homology to and evolution from peroxisomal enzymes involved in β-oxidation, e.g. SidI and SidH display significant similarity to acyl-CoA synthase and enoyl-CoA hydratase (Figs S1 and S2).

Peroxisomes are generated either *de novo* from the endoplasmatic reticulum or by PexK-dependent peroxisomal division (Hettema and Motley, 2009). PexK deficiency (strain *ΔpexK*) did not affect TAFC production (Fig. 5) which indicates that *de novo* peroxisome biogenesis from the endoplasmatic reticulum is sufficient for TAFC biosynthesis. In contrast, PexK is required for the increase of peroxisomes during growth on fatty acids and optimal β-oxidation (Valenciano *et al*., 1996) (Hynes *et al*., 2008). Deficiency in peroxisomal biogenesis, protein import or ATP import did not decrease FC production (strains *ΔpexE, pexF23, pexC::bar* and *ΔpexK*). Intriguingly, all investigated deficiencies impairing SidI localization or activity (*pexG14*, *ΔpexE/*pexG14 and *antA15*) displayed even increased FC contents (Fig. 5). Inactivation of SidI blocks mevalonate consumption for siderophore biosynthesis (Yasmin *et al*., 2011). It is likely that *N^5^*-hydroxyornithine, the common precursor for TAFC and FC, and the mevalonate precursor acetyl-CoA is redirected to FC biosynthesis.

Taken together, this study has demonstrated for the first time that particular siderophore biosynthetic enzymes are localized in peroxisomes and that this compartmentalization is evolutionary conserved. Remarkably, the peroxisomal part of siderophore biosynthesis is an example of a metabolic pathway that functions as long as all three components are localized in the same compartment independent of peroxisomes, which is indicated by the fact that siderophore production was impaired by cytoplasmic mislocalization of individual enzymes but not the complete loss of peroxisomes. The importance of iron acquisition in the pathogenicity of both plant and animal fungal pathogens has been shown in many studies (Haas *et al*., 2008). Our results indicate that peroxisomes play a crucial role in the production of virulence-determining siderophores.

## Experimental procedures

### Strains, oligonucleotides, plasmids, growth conditions

The fungal strains, plasmids and oligonucleotides used in this study are listed in Table S4, Table S5 and Table S6. Generally, *A. fumigatus* and *A. nidulans* strains were grown at 37°C in *Aspergillus* minimal medium according to Pontecorvo *et al*. (1953) containing 1% glucose as the carbon source and 20 mM glutamine as the nitrogen source. Iron-replete media (+Fe) contained 30 μM FeSO_4_. For iron-depleted conditions (−Fe), addition of iron was omitted. The final concentration for required supplements was 1 mg l^−1^ biotin, 4 mg l^−1^ p-aminobenzoic acid, 2.5 mg l^−1^ pyridoxine, 2.5 mg l^−1^ riboflavin. The bathophenanthroline disulphonate (BPS) concentration used was 200 μM. *N. crassa* was grown in standard Vogel's medium (Vogel, 1956) containing 2% sucrose as carbon source and 20 mM glutamine as the nitrogen source and 1 mg l^−1^ biotin. For iron-depleted conditions, iron was omitted.

### DNA manipulations

For extraction of genomic DNA, mycelia were homogenized and DNA was isolated according to Sambrook *et al*. (1989). For general DNA propagations *Escherichia coli* DH5α strain was used as a host.

### Generation of fungal strains expressing GFP-tagged versions of the studied enzymes

The *sidH* (AFUA_3g03410) and *sidF* (AFUA_3g03400) coding regions including introns were amplified from cosmid psidD-COS as template using primers osidHgfp1 and osidHgfp3 with add-on BglII and NotI sites for *sidH* and ogfpsidF3 and osidHgfp2 with add-on BamHI and NotI sites for *sidF* respectively. The PCR products were cloned in frame with the 5′-preceding GFP-encoding region into the BglII/NotI restriction sites of the plasmid pCAME703-AoHapX-full, replacing the original *hapX* fusion (Goda *et al*., 2005). This expression vector for the GFP-fusion protein is driven by a constitutive XAH promoter from *Chaetomium gracile*. A PTS1 lacking version of SidH was constructed with the primers osidHgfp1 and osidHgfp2 in the same manner. In order to express the *sidI* orthologue from *N. crassa*, RNA from mycelia grown for 72 h under iron starvation was transcribed into cDNA (SuperScript II Kit, Invitrogen). This cDNA was then used as a template for PCR-amplification of the *N. crassa sidI* coding region excluding introns using the primers oNcSidI-1/oNcSidI-2 containing add-on BglII and /NotI sites. The resulting fragment was cloned into the BglII/NotI sites of the plasmid pCAME703-AoHapX-full resulting in plasmid pGFP–NcSidI. The resulting plasmids pGFP–SidH, pGFP–SidF, pGFP–SidH^ΔPTS1^ and pGFP–NcSidI were then used to transform the respective *A. fumigatus* and *A. nidulans* strains. Transformation of *Aspergillus* spp. was carried out as described by Tilburn *et al*. (1995). The selection was ensured by co-transformation with the plasmid pSK275, which carries the pyrithiamine resistance gene *ptrA* using 0.1 μg ml^−1^ pyrithiamine (Sigma). The screening for transformants was performed by PCR (ogfp1/ oAf538RAC1-f for *gfp-sidH* and ogfp4/oAf538-AT1-r for *gfp-sidF*), TAFC production and GFP-fluorescence.

To visualize localization of SidI, a *sidI–gfp* gene fusion encoding a fusion of SidI C-terminus with the enhanced green fluorescent protein (EGFP) was constructed. Therefore, a 1.2 kb fragment encoding the C-terminal region of SidI was amplified using oligonucleotides oAfsidI-1 and oAfsidI-2, thereby replacing the *sidI* stop codon with a BamHI site. A 2.2 kb SmaI–SacI GFP-encoding fragment was subcloned from plasmid pUCGH (Langfelder *et al*., 2001) into the compatible EcoRV–SacI sites of plasmid pGEM-5zf(+) (Promega), yielding plasmid pGfp. The pGfp and the PCR product were digested with SphI and BamHI respectively. Both the insert and the vector were made blunt ended using Klenow fragment and ligated to give plasmid pSidI–Gfp. A 2.2 kb BssHII fragment of *ptrA* gene from psk275 was inserted into the BssHII site of pGem-sidI, yielding pSPGfp. pSPGfp was introduced into the *wt* strain by protoplast transformation. Pyrithiamine-resistant transformants containing the SidI–GFP in-frame fusion of the *sidI* and EGFP-encoding genes (*sidIgfp* strain) were selected and used for the subcellular localization of SidI. Single homologous recombination was confirmed by Southern blot analysis of XhoI digested DNA.

To gain a plasmid carrying the gene plus promoter encoding the entire SidI–GFP fusion protein, a 5 kb fragment was amplified from genomic DNA of the sidI^gfp^ strain using oligonucleotides oAfsidIgfp1 and oAfsidIgfp2 with add-on EcoRV and ClaI sites respectively. The PCR product was cloned into the EcoRV/ClaI site of the plasmid pphleo. The resulting plasmid pSidI–gfp2 carries the endogenous promotor with *sidI–gfp* codons and was transformed in *A. nidulans* strains. Phleomycin-resistant transformants containing the SidI–GFP were selected and used for the subcellular localization of SidI.

For the colocalization studies of GFP–SidH and RFP–PTS1, the RFP–PTS1-encoding plasmid pSK379–RFP–PTS1 was integrated into the GFP–SidH producing *A. fumigatus* mutant by co-transformation with the plasmid pphleo, which confers phleomycine resistance. Transformants were screened via resistance to pyrithiamine and phleomycin.

### Analysis of siderophores

Analysis of siderophore was carried out by reversed phase HPLC as described previously (Oberegger *et al*., 2001). To quantify extracellular or intracellular siderophores, culture supernatants or cellular extracts were saturated with FeSO_4_ and siderophores were extracted with 0.2 volumes of phenol. The phenol phase was separated and subsequent to addition of five volumes of diethylether and one volume of water, the siderophore concentration of the aqueous phase was measured photometrically using a molar extinction factor of 2996/440 nm (M^−1^ cm^−1^) for TAFC and 2460/434 nm (M^−1^ cm^−1^) for FC.

### Fluorescence microscopy

For confocal microscopy strains were grown in glass bottom dishes (MatTec) with supplemented minimal media for 17 h at 37°C. Images were taken on a confocal laser scanning microscope (SP5, Leica) equipped with a 63×/1.40 oil immersion objective. Z series of optical sections were obtained and projected along the z axis to obtain a general view of the specimen. The acquisition software was LAS AF software (Leica Microsystems). Images were processed using ImageJ (http://rsbweb.nih.gov/ij/).

### Computational analysis

The following databases were used for gene comparisons, NCBI: http://www.ncbi.nlm.nih.gov/; Broad Institute: http://www.broadinstitute.org/. PTS1 predictions were performed using the general function of the PTS1 Predictor (http://mendel.imp.ac.at/jspcgi/cgi-bin/pts1/pts1.cgi). PTS2 motifs were identified using a PTS2 finder (http://www.peroxisomedb.org/diy_PTS2.html).
