# Supplementary material for: Fungal siderophore biosynthesis is partially localized in peroxisomes
Source: Mol Microbiol. 2013 Apr 26;88(5):862–75. doi: 10.1111/mmi.12225 (PMC3709128; doi:10.1111/mmi.12225)
Supplement: Supplementary file 1 [file mmi0088-0862-SD1.pdf]

Online supplemental material

Table S1. Genomic organization of the siderophore biosynthetic genes *sidH, sidF, sidI, sidD, sidC* and *sidA* in various fungal species. Genes that are colocalized in the genome, i.e. organized in gene clusters, are marked in grey or black, respectively. Gey and black represent two different gene clusters. The gene accession numbers encoding orthologs to SidI, SidH and SidF correspond to the proteins listed in Table 1 and Table S2. Abbreviations: E, *Eurotiomycetes*, L; *Leotiomycetes*; S, *Sordariomycetes*; D, *Dothideomycetes*; B, *Basidiomycota*.

sidH	***sidF***	***sidD***	***sidI***	***sidC***	***sidA***		
AFUA_3G03410	AFUA_3G03400	AFUA_3G03420	AFUA_1G17190	AFUA_1G17200	AFUA_2G07680	*Aspergillus fumigatus*	*E*
NFIA_005580	NFIA_005570	NFIA_005590	NFIA_008180	NFIA_008170	NFIA_083410	*Neosartorya fischeri*	
AO090103000168	AO090103000169	AO090103000167	AO090023000529	AO090023000528	AO090011000926	*Aspergillus oryzae*	
AN6235	AN6234	AN6236	AN0609	AN0607	AN5823	*Aspergillus nidulans*	
An03g03550	An03g03540	An03g03520	An06g01320	An06g01300	An05g00220	*Aspergillus niger*	
ACLA_061010	ACLA_061020	ACLA_061000	ACLA_018340	ACLA_079690	ACLA_079680	*Aspergillus clavatus*	
TSTA_100270	TSTA_100280	TSTA_100300	TSTA_041590	TSTA_090280	TSTA_090270	*Talaromyces stipitatus*	
Pc22g20370	Pc22g20380	Pc22g20400	Pc21g23730	Pc13g05250	Pc13g05260	*Penicillium chrysogenum*	
HCBG_04177	HCAG_08322	HCBG_09033	HCAG_08321	HCBG_04660	HCAG_08320	*Ajellomyces capsulatus*	
BC1G_10565	BC1G_10564	BC1G_10567	BC1G_14541*	BC1G_03511	BC1G_03506	*Botryotinia fuckeliana*	*L*
SS1G_04249	SS1G_04248	SS1G_04250	SS1G_13961	SS1G_06185	SS1G_03696	*Sclerotinia sclerotiorum*	
FG03746	FG03745	FG03747	FG04333	FG11026	FG05872	*Gibberella zeae*	*S*
CHGG_09472	CHGG_09473	CHGG_09475	CHGG_10903	CHGG_02251	CHGG_09542	*Chaetomium globosum*	
PODANS72p146	PODANSg2355	PODANSg524	PODANSg2356	PODANSg3444	PODANSg3445	*Podospora anserina*	
NCU06960	NCU06062	NCU08441	NCU06063	NCU07119	NCU07117	*Neurospora crassa*	
MGG_14970	MGG_08289	MGG02351	MGG_08288	MGG_12175	MGG_04212	*Magnaporthe oryzae*	
SNOG_00297	SNOG_14362	SNOG_14368	SNOG_14361	SNOG_14834	SNOG_14364	*Phaeosphaeria nodorum*	*D*
UM01433	UM01432	UM01434	UM04803	UM05165	UM10188	*Ustilago maydis*	*B*

Table S2. Comparison of siderophore biosynthetic genes of *A. fumigatus* and *A. nidulans*.

gene	gene locus[a]	*A. nidulans* ortholog[a] (gene locus)	% identity (similarity)[b]	length[a]	proposed function
sidA	AFUA_2G07680	AN5823.4	77 (86)	498	N^5-ornithine-monooxygenase (Eisendle et al., 2003)
sidI	AFUA_1G17190	AN0609.4	70 (77)	590	TAFC biosynthetic acyl-CoA ligase, PTS2 (Yasmin et al., 2011)
sidH	AFUA_3G03410	AN6235.4	84 (90)	270	TAFC biosynthetic enoyl-CoA hydratase, PTS1 (Yasmin et al., 2011)
sidF	AFUA_3G03400	AN6234.4	79 (89)	462	TAFC biosynthetic transacylase PTS1 (Schrettl et al., 2007)
sidD	AFUA_3G03420	AN6236.4	64 (78)	2086	fusarinine C NRPS (Schrettl et al., 2007)
sidG	AFUA_3G03650	AN8539.4	57 (72)	202	TAFC biosynthetic transacetylase (Schrettl et al., 2007)
sidL	AFUA_1G04450	AN10080.4	62 (75)	503	FC biosynthetic transacetylase (Blatzer et al., 2011)
sidC	AFUA1G17200	AN0607.4	55 (71)	4763	Ferricrocin NRPS (Schrettl et al., 2007)

[a] predicted gene and length of protein based on gene annotations (http://www.broad.mit.edu/annotation/genome/aspergillus_group/MultiHome.html)
[b] BlastP (Altschul *et al.*, 1990) of *A. fumigatus* protein against predicted *A. nidulans* sequence

Table S3. Comparison of peroxins of *A. fumigatus* and *A. nidulans.*

Gene	gene locus[a]	***deletion strain***	***A. fumigatus ortholog***[a]	% identity (similarity)[b]	length[a]	proposed function
pexC	AN2281.4	*pexC::bar*	AFUA_5G06300	70 (82)	530	peroxisome membrane biogenesis
pexE	AN10215.4	Δ*pexE*	AFUA_8G05240	71 (76)	655	PTS1 protein import receptor
pexF	AN2925.4	*pexF23*	AFUA_3G08000	78 (87)	1476	AAA ATPase, Pex5 recycling
pexG	AN0880.4	*pexG14*	AFUA_1G15400	85 (92)	355	PTS2 protein import receptor
pexK	AN1921.4	Δ*pexK*	AFUA_6G07740	87 (96)	235	peroxisome proliferation
antA	AN0257.4	*antA15*	AFUA_1G03440	83 (91)	336	peroxisomal ATP carrier

[a] predicted gene and length of protein based on gene annotations (http://www.broad.mit.edu/annotation/genome/aspergillus_group/MultiHome.html)
[b] BlastP (Altschul et al., 1990) of *A. fumigatus* protein against predicted *A. nidulans* sequence

Table S4. Strains used in this study

	genotype	reference
Aspergillus fumigatus		
ATTCC46645	*wild type (wt)*	American type culture
ΔsidH	*wt, sidH::hph/tk*	(Yasmin et al., 2011)
ΔsidF	*wt; sidF::hph*	(Schrettl et al., 2007)
ΔsidI	*wt; sidI::hph/tk*	(Yasmin et al., 2011)
sidIgfp	*wt; sidI::sidI-gfp, ptrA*	this study
gfp-sidH	*ΔsidH, gfp-sidH(p), ptrA(p)*	this study
gfp-sidF	*ΔsidF, gfp-sidF(p), ptrA(p)*	this study
gfp-sidHΔPTS1	*ΔsidH, gfp-sidHΔPTS1(p), ptrA(p)*	this study
gfp-sidH/rfp-pts1	*gfp-sidH,* rfp-pts1(p), *phleo(p)*	this study
Aspergillus nidulans		
WGTRAN, wt	*argB2::pTran, argB; bgA0; biA1*	(Brakhage & Turner, 1992)
pexC::bar	*biA1 niiA4 pyroA4 pexC::bar*	(Hynes et al., 2008),
ΔpexE	*yA1, su-ad ad E20, pyroA4, areA102, riboB2,*	(Hynes et al., 2008),
pexF23	*yA1, pabaAI, PexF(6)23*	(Hynes et al., 2008), BU223
pexG14	*yA1, pabaAI, PexG(7)14*	(Hynes et al., 2008), BU414
ΔpexK	*yA1, su-ad ad E20, pyroA4, riboB2, areA102*	(Hynes et al., 2008),
pexK-gfp	*nkuAΔ pyroA4, pexK::gfp(PyroA+)*	(Hynes et al., 2008),
pexCc	*pexC::bar, pI4(pyroA+) pexC*	Hynes unpublished,
pexEc	*ΔpexE, pI4(pyroA+) pexE*	Hynes unpublished,
ΔpexE/pexF23	*yA1, pabaAI, pyroA4 , PexG14, pexE(5)Δ(ribo+)*	Hynes unpublished,
wt gfp-sidH	*wt, gfp-sidH(Af)(p); ptrA(p)*	this study
ΔpexE gfp-sidH	*ΔpexE gfp-sidH(Af)(p), ptrA(p)*	this study
pexG14 gfp-sidH	*pexG14 gfp-sidH(Af)(p), ptrA(p)*	this study
wt gfp-sidI(Nc)	*wt, gfp-sidI(Nc)(p), ptrA(p)*	this study
ΔpexE gfp-sidI(Nc)	*ΔpexE gfp-sidI(Nc)(p), ptrA(p)*	this study
ΔpexE/pexF23	*yA1, pabaAI, pyroA4 , PexG14, pexE(5)Δ(ribo+)*	Hynes unpublished,
antA15	*yA1, pabaAI, antA15*	(Hynes et al., 2008),
wt sidI-gfp	*wt, sidI-gfp(Af)(p); phleo(p)*	this study
pexG14 sidI-gfp	*pexG14 sidI-gfp(Af)(p), phleo(p)*	this study
Neurospora crassa		
74-OR23-1A(FGSC	*wild type (A)*	American type culture

Table S5. Plasmids used in this study

Name	purpose	reference
psidD-COS	cosmid carrying *sidD* and adjacent *sidH* and *sidF*	(Schrettl et al., 2007)
pPhleo	the phleomycin resistance marker gene *ble* in bluescript	(Punt *et al.*, 1987)
pUCGH	carrying EGFP-encoding gene	(Langfelder et al., 2001)
pCAME703-AoHapX-full	expression vector for N-terminal GFP tagging	(Goda *et al.*, 2005)
pSK275	pyrithiamine resistance gene *ptrA*	(Kubodera *et al.*, 2000)
pGfp	EGFP-encoding gene in pGEM-5zf(+)	this study
pSidI-Gfp	C-terminal EGFP tagged *A. fumigatus* SidI truncation in pGEM-5zf(+)	this study
pSPGfp	C-terminal EGFP tagged *A. fumigatus* SidI truncation and resistance gene ptrA	this study
psidH^{c}	functional *sidH* copy for complementation	this study
pGFP-SidH	expression plasmid encoding N-terminal GFP-tagged *A. fumigatus* SidH	this study
pGFP-SidHΔPTS1	expression plasmid encoding N-terminal GFP-tagged *A. fumigatus* SidH lacking PTS1	this study
pGFP-SidF	expression plasmid encoding N-terminal GFP-tagged *A. fumigatus* SidF	this study
pGFP-NcSidI	expression plasmid encoding N-terminal GFP-tagged *N. crassa* SidI	this study
pSidI-Gfp2	Expression plasmid encoding C-terminal GFP-tagged *A. fumigatus* SidI (full length)	this study
pSK379-RFP-PTS1	Expression plasmid encoding RFP-PTS1	(Beck & Ebel, 2013)

Table S6. Oligonucleotides used in this study.

primer	sequence 5' to 3'
oAf538RAC1-f	CAC TGT GGC TTT CGC TTC
oGFP1	GAC CAC ATG AAG CAG CAC
ogfp4	AAC CAC TAC CTG AGC AAC
osidHgfp1	ACA AGA TCT TCA ACA GAA GCA CAT CCA AC
osidHgfp2	CAC GCG GCC GCT CAC CTC CAC TGT GGC TTT CG
osidHgfp3	CTG GCG GCC GCT TCA CAA CTT AGA CGG CC
ogfpsidF2	ATA GCG GCC GCT TAC AGC TTT GCA TCC CAC
ogfpsidF3	ATA GGA TCC GCG ACT CAA AGC AGC ACC
oAf$_{538}$AT1-r	AGT TTT GAG CGA GAG GGG
oAfsidI-1	CAG TGG ATC CTT TTT CCT TTA TTG CTG CC
oAfsidI-2	CTA CGG ATG TCT GCA ACC
oNcSidI-1	AAT AAT AGA TCT TCA CCG GCG TTG GTA
oNcSidI-2	TTA GCG GCC GCT CCA TCT TCT TCC CTC AG
oAfsidIgfp1	ATA GAT ATC AGC CGG ACA AGA AAC AAG
oAfsidIgfp2	TAT ATC GAT GTT TGT GGT TTC CGT TGG

Introduced restriction sites are underlined.

Table S7. Homologs of *Basidiomycetes* to *A. fumigatus* SidF identified by a BlastP search (http://www.ncbi.nlm.nih.gov/sutils/genom_table.cgi?organism=fungi).

**Protein**		**Score (Bits)**	**E Value**
XP_001731607.1	[*Malassezia globosa*]	205	3e-59
XP_757579.1	[*Ustilago maydis*]	182	1e-50
EGO03326.1	[*Serpula lacrymans var. lacrymans*]	173	9e-48
XP_003035019.1	[*Schizophyllum commune*]	171	4e-47
XP_003322498.1	[*Puccinia graminis f. sp. tritici*]	163	1e-44
EGG01975.1	[*Melampsora larici-populina*]	158	1e-43
XP_002910369.1	[*Coprinopsis cinerea okayama*]	155	1e-40
XP_756191.1	[*Ustilago maydis*]	147	4e-38

Figure S1. Similarity of *A. fumigatus* SidI with proven and putative Acyl-CoA synthases from *A. nidulans* (Reiser *et al.*, 2010). FaaB, FatA, FatB, FatC and FatD have been shown to, while PcsA is predicted to, localize to peroxisomes (A) BlastP search results (http://www.ncbi.nlm.nih.gov/blast/) using *A. fumigatus* SidI as bait; functionally proven acyl-CoA synthases are in bold; (B) Phylogenetic tree (http://www.genome.jp/tools-bin/clustalw); (C) Alignment of *A. fumigatus* SidI and the peroxisomal *A. nidulans* acyl-CoA synthase FatD.

A

Protein	Score (Bits)	E-Value
SidI-AN0609(XP_658213.1)	804	0.0
FatD-AN4397(XP_662001.1)	164	3e-44
FatB-AN5877(XP_663481.1)	97.1	2e-21
**FacA-AN5626(XP_663230.1)**	**86.7**	**5e-18**
FatA-AN5192(XP_662796.1)	87.4	3e-18
**PcsA-AN5833(XP_663437.1)**	**80.9**	**3e-16**
**FaaB-AN8280(XP_681549.1)**	**57.8**	**6e-09**
FaaA-AN6014(XP_663618.1)	56.6	1e-08
FatC-AN6649(XP_664253.1)	50.4	9e-07

B

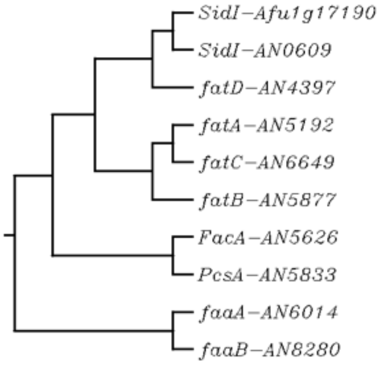

C

```
Identities = 147/523 (28%), Positives = 236/523 (45%), Gaps = 55/523 (11%)

AfSidI  66   TYTDLKDEADRVARGLLAMGIQKGDRIGIMAGNCEQYISVFFAAARVGAILVVLNNTYTP  125
             TY  L          L  +G+  G  + +   N  ++I  F AA+   AI   LN  Y
AnFatD  28   TYAQLHSHISAFQEKLAKLGVGHGAAVNLALINSYEFIVGFLAASWQRAIAAPLNPAYKQ  87

AfSidI  126  SELYYALGHTDCRLLFLTPRIGRHSLEEVLAKLGPRPKEQGTSSALEEIIILRGQYSGFS  185
              E  + +      L+ L PR       E  A+ GP       +    +  I    ++G
AnFatD  88   DEFEFYIDDLSSTLV-LIPR-------ESYAQNGPAV----LAGRKYQAAIAECYWNGTE  135

AfSidI  186  TYEHVIQRGLPLPSHALQDREAELHSTDVCNLQFTSGSTGNPKAAMLTHHNLVNNSRFIG  245
                 V + G       +  + A+    D+  +  TSG+TG PKA  LTH NL    R I
AnFatD  136  VVLDVKELGKLKGKGDIGVQTAQ--PDDIALVLHTSGTTGRPKAVPLTHKNLTTTMRNIQ  193

AfSidI  246  DRMNLTSFDILCCPPPLFHCFGLVLGMLAVVTHGSKIIFPSETFDPTAVLHAISDEKCTA  305
                 LT  D      PLFH  GL+   LA +  G  +I P++ F          + K
AnFatD  194  ATYKLTPQDRTYLVMPLFHVHGLLAAFLAPLASGGSVIVPTK-FSAHQFWSDFIEYKANW  252

AfSidI  306  LHGVPTMFEAILSLPKP-PNFDCSNLRTGIIAGAPVPRPLMKRLLEELNMT---EYTSSY  361
                VPT+ + +L  P P P      +R+          PL  +  ++L  T       +Y
AnFatD  253  YSAVPTIHQILLKSPLPNPIPQIRFIRS-------CSSPLSPKTFQDLEKTLNAPVLEAY  305

AfSidI  362  GLTEASPTCFNALTTDSI---ERRLTTVGKVMPHAKAKIIDTQGHIVPIGQRGELCIAGY  418
              +TEA+    + +T++ +   +R+  +VG +    + KI+D  G+ VP G   E+C+ G
AnFatD  306  AMTEAA----HQMTSNPLPPAKRQPGSVG-IGQGVEIKILDQSGNEVPQGHEAEICVRGE  360

AfSidI  419  QLTKGYWNNPEKTAEALITDSDGVTWLKTGDEAIFDEEGYCSITGRFKDIIIRGGENIYP  478
              +TKGY NNP     +   D     + +TGD+   D +GY  ITGR K++I +GGE I P
AnFatD  361  NVTKGYLNNPAANKSSFTKDG----FFRTGDQGKKDPDGYVIITGRIKELINKGGEKISP  416

AfSidI  479  LEIEERLAAHPAIEVASVIGIPDQ-KYGEVVGAFLALAADVSARPSDEELRAWTRETLGR  537
             +E++  L  +P +  A    IPD   YGE +GA + L +  +A  +++EL++W +E L +
AnFatD  417  IELDNTLLQNPNVGEAVCFAIPDPGHYGEDIGAAVVLKSGQNA--TEDELKSWVQEKLAK  474

AfSidI  538  HKAPQYFFVFGEEGVDRTIPVTGSGKVRKVDLRKIAASVLERR  580
              K P+           + IP T +GK+++   RK+A ++L+ +
AnFatD  475  FKTPK-----------QQIPKTATGKIQR---RKVAEAMLKPK  503
```

Figure S2. Alignment of *A. fumigatus* SidH and the mitochondrial localized enoyl-CoA hydratase EchA from *A. nidulans* that is involved in ß-oxidation (Maggio-Hall & Keller, 2004). Homologs with PTS1 motifs have been postulated to be involved in peroxisomal ß-oxidation (Maggio-Hall & Keller, 2004).

```
BlastP: Score = 228, E-value = 2e-73
Identities = 123/279(44%), Positives = 167/279(59%), Gaps = 15/279(5%)

Af SidH  1    MSTEAHPTVQGCLVSFPTPHI-------LLLTLNRPEKRNCISLATSAEIQRLWTWFDTQ  53
              + + A P+ +  L S P P +       +++TLNRP+  N +S     E+    + +D
An EchA  23   LYSSAAPSYEHILTSTPKPGVGLNTDFTVIVTLNRPKALNALSSPLFKEVNDALSKYDES  82

Af SidH  54   PALYVAIITGTGESFCAGADLKEWNDLN-ARGITNEMTAP--GLAGLPRRRGSKPIIAAV  110
                +   IITG+ ++F AGAD+KE   L  A   +N   AP   LA   R    KP+IAAV
An EchA  83   KDIGAIIITGSEKAFAAGADIKEMAPLTFASAYSNNFIAPWSHLANSIR----KPVIAAV  138

Af SidH  111  NGYCLGGGFEMVANCDIVVASENATFGLPEVQRGIAAVAGSLPRLVRVLGKQRAAEIALS  170
              +G+ LGGG E+   CDI+  + +ATFG PE++ G+   AG   RL   +GK +A E+ L+
An EchA  139  SGFALGGGCELALMCDIIYCTASATFGQPEIKLGVIPGAGGSQRLTAAVGKSKAMELILT  198

Af SidH  171  GLSFSASQLERWGLVNRVVE--HDQLLATAVEIATAISRNSPDSVRVTMEGLHYGWEMAS  228
              G +FS  +   WG+  +VV+   ++LL  AV+ A  I+  S  +     E ++   ++
An EchA  199  GKNFSGKEAGEWGVAAKVVDGGKEELLEEAVKTAETIAGYSRVATVAAKEVVNKSQDLGV  258

Af SidH  229  VE--EASSALV-------DQWYAKLMA-------GENFHEGVRAFVEKRKPQW  265
               E  E    L        DQ   + ++       G +  +G+ AF EK+KPQW
An EchA  259  REGVEYERRLFHGLFGSQDQKIGRFLSLYHYSKNGTDGEKGMTAFAEKKKPQW  311
```
